# Evolution of DELLA function and signaling in land plants

**DOI:** 10.1111/ede.12365

**Published:** 2021-01-11

**Authors:** Alexandros Phokas, Juliet C. Coates

**Affiliations:** ^1^ School of Biosciences University of Birmingham Edgbaston Birmingham UK

## Abstract

DELLA proteins are master growth regulators that repress responses to a group of plant growth hormones called gibberellins (GAs). Manipulation of DELLA function and signaling was instrumental in the development of high‐yielding crop varieties that saved millions from starvation during the “Green Revolution.” Despite decades of extensive research, it is still unclear how DELLA function and signaling mechanisms evolved within the land plant lineage. Here, we review current knowledge on DELLA protein function with reference to structure, posttranslational modifications, downstream transcriptional targets, and protein–protein interactions. Furthermore, we discuss older and recent findings regarding the evolution of DELLA signaling within the land plant lineage, with an emphasis on bryophytes, and identify future avenues of research that would enable us to shed more light on the evolution of DELLA signaling. Unraveling how DELLA function and signaling mechanisms have evolved could enable us to engineer better crops in an attempt to contribute to mitigating the effects of global warming and achieving global food security.

## Research Highlights

- *DELLA* genes first appeared in the common ancestor of land plants and underwent two major duplications during land plant evolution.
- DELLAs repress gibberellin responses in vascular plants but their function in nonvascular plants remains elusive.

## Graphical abstract

**DELLA protein evolution in land plants**. DELLA protein‐encoding genes first appeared in the ancestor of land plants. There have been subsequent gene duplications in the vascular plant ancestor and in eudicot flowering plants, with gene losses in ferns and some flowering plant families. The transactivation ability of DELLA proteins originated in the ancestor of land plants, while canonical gibberellin (GA) signaling arose in the ancestor of vascular plants. DELLA protein regulation is well‐researched in flowering plants but more research is required to determine whether regulatory mechanisms are conserved in early‐diverging lineages.

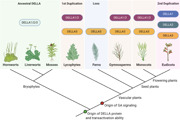

## 1 INTRODUCTION

One of the most important innovations of the 20th century that led to the expansion of modern‐day agriculture was the development of novel cultivation methods and high‐yielding crop varieties, an initiative that has been termed the “Green Revolution” (Peng et al., 1999). The “Green Revolution” took place in the 1960s and 1970s, and was characterized by increased application of fertilizers and pesticides as well as the production of semi‐dwarf wheat and rice varieties with higher grain yields, reduced lodging, and higher tolerance to large amounts of fertilizer (Gale & Youssefian, 1985; Hedden, 2003). As a consequence of the “Green Revolution,” crop yields in developing countries increased initially by 21% and, after the 1970s, by 50%, global food prices fell significantly, and millions of people were saved from starvation (Evenson & Gollin, 2003). Subsequent research led to the identification of the alleles of genes responsible for conferring those semi‐dwarf phenotypes. Among these were alleles of the the wheat genes *REDUCED HEIGHT* (*TaRHT*)‐*B1b* and *TaRHT*‐*D1b*, which encode DELLA proteins (Peng et al., 1999).

## 2 DELLA PROTEINS: REPRESSORS OF VASCULAR PLANT GIBBERELLIN RESPONSES

DELLA proteins are master growth repressors belonging to the GRAS (named after GIBBERELLIN INSENSITIVE [GAI], REPRESSOR OF GA1‐3 [RGA], and SCARECROW [SCR]) family of putative transcriptional regulators (Peng et al., 1997; Pysh et al., 1999). They are present exclusively in land plants and they vary in numbers between species (Hernández‐García et al., 2019). Some species have just a single DELLA protein, such as rice (SLENDER RICE1, *Os*SLR1), barley (SLENDER1, *Hv*SLN1) and tomato (PROCERA, *Sl*PRO; Gubler et al., 2002; Ikeda et al., 2001; Jasinski et al., 2008), while others have more than one, for example, *Arabidopsis thaliana* has five: *At*GAI1, *At*RGA1, *At*RGA‐LIKE1 (*At*RGL1), *At*RGL2, and *At*RGL3, which have distinct and overlapping functions (Dill & Sun, 2001; Peng & Harberd, 1993; Sánchez‐Fernández et al., 1998; Silverstone et al., 1997).

DELLA proteins get their name from five conserved amino acids (aspartic acid, glutamic acid, leucine, leucine, and alanine), present in their N‐terminal domain. This domain is important for their regulatory function and is absent in other GRAS family proteins such as *At*SCR. The primary role of DELLA proteins in vascular plants is to repress responses to a group of structurally related plant hormones, gibberellins (GAs), which promote many major developmental responses in plants, such as germination, stem elongation, leaf expansion, and flowering (Olszewski et al., 2002). As DELLAs do not possess a DNA‐binding domain, they exert their growth repression by interacting mainly with transcription factors that regulate these responses (Feng et al., 2008).

## 3 THE EVOLUTION OF DELLA PROTEINS IN LAND PLANTS: AN OVERVIEW

**Figure 1:**
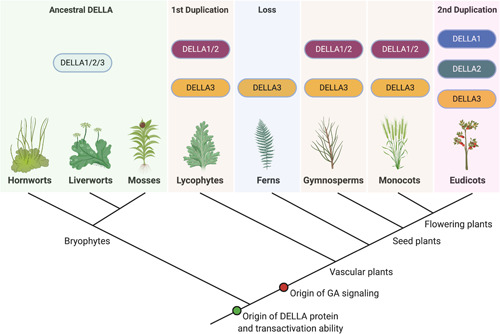
The evolution of DELLA proteins in land plants. DELLA protein‐encoding genes (DELLA1/2/3 clade) appeared in the ancestor of land plants and were maintained in bryophytes without any major duplications. The first major duplication of DELLA‐encoding genes occurred in the ancestor of vascular plants, giving rise to the DELLA1/2 and DELLA3 clades, which were maintained in gymnosperms and monocot flowering plants. In ferns, the DELLA3 clade was retained, but DELLA1/2 was lost. The second major duplication of DELLA‐encoding genes happened in eudicot flowering plants, where the DELLA1/2 clade gave rise to the DELLA1 and DELLA2 clades, while DELLA3 was retained. Further duplications and/or losses have also occurred in several flowering plant species or families. DELLA transactivation ability originated in the ancestor of land plants and canonical gibberellin (GA) signaling in the ancestor of vascular plants (figure created with BioRender.com; hornwort icon drawn by Debbie Maizels) [Color figure can be viewed at wileyonlinelibrary.com]

The evolution of DELLA proteins in land plants has attracted the attention of researchers for over a decade now. With the recent increase in the availability of genomic and transcriptomic data, we are now starting to get a clearer picture of how DELLA proteins evolved in the different land plant lineages. Independent phylogenetic analyses have suggested that two duplication events have occurred in the history of *DELLA* protein‐encoding genes: the first one in the ancestor of vascular plants and the second one in eudicot flowering plants (Figure 1; Hernández‐García et al., 2019; Van De Velde et al., 2017). Consequently, nonvascular plants (bryophytes) possess a single DELLA clade, termed DELLA1/2/3 (Hernández‐García et al., 2020), vascular plants (excluding eudicots) possess a DELLA1/2 clade and a DELLA3 clade, and eudicots have three DELLA clades termed DELLA1 or RGA, DELLA2 or RGL, and DELLA3 or DGLLA (Figure 1; Hernández‐García et al., 2019; Van De Velde et al., 2017).

Interestingly, ferns appear to lack the DELLA1/2 clade and several flowering plant species have lost clades or have undergone further DELLA duplications (Hernández‐García et al., 2019). For example, in tomato, a eudicot flowering plant, the DELLA1 and DELLA3 clades have been lost, while in rice, a monocot flowering plant, DELLA3 clade proteins have lost their N‐terminal DELLA domains, but have retained their ability to repress growth (Itoh, Shimada, et al., 2005; Jasinski et al., 2008). It has been suggested that rice DELLA3 clade proteins may be part of a mechanism that evolved to inhibit growth under certain conditions where levels of the rice DELLA1/2 clade protein (*Os*SLR1) are low (Itoh, Shimada, et al., 2005; Van De Velde et al., 2017). Whether this is a common property among DELLA3 clade proteins remains unknown.

In addition, the increased number of DELLA proteins found in some flowering plant species does not correlate with increased diversity of DELLA functions, as single DELLA proteins in rice or tomato perform the same functions as the five *Arabidopsis* DELLAs collectively (Blázquez et al., 2020). Instead, as mentioned earlier, it appears that the diversification of DELLA functions in species with multiple DELLA proteins, such as *Arabidopsis*, is a consequence of the diversification in their expression patterns, rather than the ability of different DELLAs to interact with different partners (Gallego‐Bartolomé et al., 2010). This conclusion is based on the fact that (i) transcription factors or regulators that interact with DELLA proteins mostly do not discriminate between the different DELLA proteins within a species (e.g., Gallego‐Bartolomé et al., 2010; Lantzouni et al., 2020) and that (ii) DELLAs such as *At*RGA1 and *At*RGL2, which mostly regulate hypocotyl elongation and germination, respectively, can perform exchangeable functions when expressed under each other's promoter (Gallego‐Bartolomé et al., 2010). Under this hypothesis, DELLA proteins would have started with a general growth repressive function, which would have then been refined in a tissue‐specific manner, for example, the repression of germination by *At*RGL2 in *Arabidopsis* seeds.

## 4 LIFTING THE GROWTH REPRESSIVE FUNCTION OF DELLAS VIA GA SIGNALING

**Figure 2:**
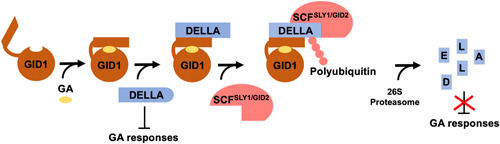
DELLA proteins repress gibberellin (GA) responses in vascular plants and DELLA repression is lifted by GAs via DELLA degradation. The GA receptor, GID1, perceives GA and secures it within its GA‐binding pocket using its N‐terminal lid‐like structure. The GA–GID1 complex can then sequester DELLA protein, enabling a SKIP1‐CUL1‐F‐box (SCF) E3 ligase complex containing an F‐box protein such as SLEEPY1 (SLY1) in *Arabidopsis thaliana* or GID2 in rice, to induce DELLA polyubiquitination and subsequent degradation via the 26S proteasome. DELLA degradation then releases GA responses from repression [Color figure can be viewed at wileyonlinelibrary.com]

**Figure 3:**
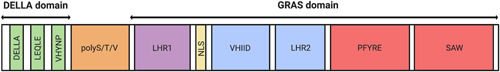
DELLA protein domain structure. The N‐terminal DELLA domain contains the DELLA, LEQLE, and VHYNP motifs required for interaction with GA–GID1 and GA‐dependent degradation. The C‐terminal GRAS domain consists of two leucine heptad repeat (LHR) subdomains: LHR1, which is required for protein–protein interactions mediating repression on GA responses and LHR2, which along with the VHIID subdomain are required for DELLA interaction with SLEEPY1 or GID2. The C‐terminal PFYRE and SAW subdomains are involved in repression on GA responses and interaction with GID1. The GRAS domain also contains a nuclear localization signal (NLS) motif. The N‐terminal and C‐terminal domains are linked with a homopolymeric region rich in serine, threonine, and valine (polyS/T/V), which is involved in posttranslational modifications (figure created with BioRender.com) [Color figure can be viewed at wileyonlinelibrary.com]

DELLA‐induced repression of vascular plant GA hormone responses can be overcome by GAs themselves in a dose‐dependent manner (Itoh et al., 2002). GAs are perceived by GA‐INSENSITIVE DWARF1 (GID1) receptors, which bind GAs in a pocket‐like structure (Figure 2; Murase et al., 2008; Ueguchi‐Tanaka et al., 2005). This interaction triggers a GID1 N‐terminal extension to fold back and form a lid‐like structure that secures GA into the GID1 pocket, preventing GA from coming into contact with DELLAs (Murase et al., 2008; Shimada et al., 2008; Ueguchi‐Tanaka et al., 2005). The GA–GID1 complex formed is then able to sequester DELLA proteins in the nucleus, an interaction that requires the N‐terminal DELLA domain (Figure 3; Ueguchi‐Tanaka et al., 2005, 2007). Binding of the DELLA domain to the GID1 lid stabilizes the GA–GID1 complex further and presumably triggers a conformational change in the C‐terminal GRAS domain of DELLA, allowing F‐box proteins, such as *Os*GID2 in rice, or SLEEPY1 (*At*SLY1) in *Arabidopsis*, which form part of a SKIP1‐CUL1‐F‐box (SCF) E3 ligase complex, to bind DELLAs and polyubiquitinate them (Hirano et al., 2010; McGinnis et al., 2003; Sasaki et al., 2003; Ueguchi‐Tanaka et al., 2007). Polyubiquitinated DELLAs are then degraded by the 26S proteasome and repression on GA responses is lifted (Fu et al., 2002; Sasaki et al., 2003).

DELLA repression can also be overcome in a proteolysis‐independent manner via interaction with the GA–GID1 complex, which reduces DELLA repression in *Atsly1* mutants where *At*GID1 has been overexpressed (Ariizumi et al., 2008). Furthermore, a recent study has demonstrated that the DELLA interaction with *At*GID1 can be inhibited by the circadian clock component GIGANTEA (*At*GI), which can bind and stabilize DELLA during daytime under short‐day conditions, thus regulating the diurnal rhythmic accumulation pattern of DELLA proteins (Nohales & Kay, 2019).

## 5 DELLA PROTEIN FUNCTION AND REGULATION: IMPLICATIONS FOR EVOLUTION

DELLA proteins possess a characteristic domain structure that appears largely conserved across land plants (Hernández‐García et al., 2019). How has DELLA protein function diversified throughout land plant evolution? The evidence from flowering plants suggests that DELLAs can indirectly regulate transcription via different mechanisms involving interactions with transcription factors (e.g., de Lucas et al., 2008; Feng et al., 2008). However, the transcriptional targets of DELLA transcription factor complexes are only characterized in a few flowering plants. In addition, there are multiple ways in which DELLA proteins themselves are post‐translationally modified to regulate their function, and these have also only been characterized in flowering plants. The following sections outline the variety of known DELLA functions and regulatory mechanisms and propose ways in which the degree of their conservation across land plants can be examined. This knowledge will be relevant for understanding how DELLA function diversified from the ancestral land plant DELLA protein.

### 5.1 DELLA structure and function

DELLA proteins share a common structure comprising an N‐terminal DELLA domain and a C‐terminal GRAS domain, which are linked together by a homopolymeric region rich in serine, threonine, and valine (polyS/T/V), a site for posttranslational modifications (PTMs) that affect the stability and activity of DELLA proteins (Figure 3; Fu et al., 2002; Itoh, Sasaki, et al., 2005; Itoh et al., 2002). Plant *della* gene mutants have been widely studied over the past three decades to shed light on the precise role of the different subdomains and motifs that make up these domains. These mutants can be divided into two categories: (i) dominant gain‐of‐function mutants, which render the DELLA protein unable to be degraded and give rise to GA‐insensitive dwarf plants, and (ii) loss‐of‐function mutants lacking DELLA activity, which give rise to slender plants with constitutively activated GA responses (Peng et al., 1997; Silverstone et al., 1998). The “Green Revolution” mutants *Tarht‐B1b* and *Tarht‐D1b* belong to the former category (Peng et al., 1999).

Functional characterization of *Tarht‐B1b* and *Tarht‐D1b* revealed that they have nucleotide substitutions that generate a stop codon in the N‐terminal domain (Peng et al., 1999). Most likely due to a cryptic translation initiation site downstream of the generated stop codon, these genes give rise to active proteins lacking the DELLA domain (Peng et al., 1999). Similar mutants have also been identified in *Arabidopsis*, for example, *Atgai‐1*, which synthesizes a DELLA that lacks 17 amino acids in its N‐terminus corresponding to the DELLA domain (Peng et al., 1997). These mutants produce active truncated DELLA proteins that can no longer interact with the GA–GID1 complex, are resistant to GA‐induced degradation and are, therefore, constitutively repressing GA responses, yielding semi‐dwarf phenotypes (Dill, Jung, et al., 2001; Itoh et al., 2002).

Several lines of evidence have confirmed that the DELLA, LEQLE, and VHYNP motifs within the DELLA domain are necessary for GA‐dependent interaction with GID1 and GA‐induced degradation (Figure 3; Itoh et al., 2002; Ueguchi‐Tanaka et al., 2007). In addition, the N‐terminal DELLA domain is responsible for the transactivation activity of DELLA proteins that ultimately represses GA responses in flowering plants, and which can be suppressed by interaction with GID1 (Hirano et al., 2012). It has been demonstrated that DELLA transactivation activity is conserved at least in bryophytes and lycophytes; however, the targets of transactivation have not yet been elucidated (Hernández‐García et al., 2019).

The repressive function of DELLA proteins on GA responses by means other than transactivation has been attributed to their C‐terminal GRAS domain (Figure 3; Hirano et al., 2010). This domain is necessary for the interaction of DELLA with *At*SLY1 or *Os*GID2 and subsequent DELLA degradation (Dill et al., 2004; Muangprom et al., 2005). Complete removal of the GRAS domain or single amino acid changes in the PFYRE or SAW subdomains (Figure 3), result in the induction of a loss‐of‐function slender phenotype, indicating that the GRAS domain is responsible for growth suppression (Itoh et al., 2002). The growth repression activity of the GRAS domain is mediated by DELLA protein–protein interactions (e.g., Bai et al., 2012; de Lucas et al., 2008). These interactions have only been characterized in flowering plants; therefore, the degree of their conservation remains elusive (see Section 5.4).

DELLA interaction with *At*SLY1/*Os*GID2 requires the VHIID and LHR2 domains (Figure 3), as amino acid substitutions in those subdomains abolish the interaction, even in the presence of GID1 and GA (Hirano et al., 2010). The LHR1 subdomain is required for DELLA dimer formation and for protein–protein interactions with transcription factors regulating GA responses (Bai et al., 2012; de Lucas et al., 2008; Itoh et al., 2002).

### 5.2 DELLA PTMs

DELLA proteins are known to undergo several types of PTMs (reviewed in Blanco‐Touriñán, Serrano‐Mislata, et al., 2020), namely polyubiquitination (Sasaki et al., 2003), phosphorylation (Wang et al., 2014), glycosylation (Zentella et al., 2017) and small ubiquitin‐like modifier (SUMO)‐ylation (Conti et al., 2014). Due to its agronomic relevance, the most well‐characterized DELLA PTM is polyubiquitination, which occurs via the SCF^SLY1/GID2^ complex and is necessary for proteasomal degradation of DELLAs (Figure 2; Sasaki et al., 2003). It has been demonstrated that DELLA degradation can also be induced by the E3 ubiquitin ligase CONSTITUTIVE PHOTOMORPHOGENIC1 (*At*COP1) via ubiquitination in a GA‐independent manner upon exposure to warm temperatures or shade (Blanco‐Touriñán, Legris, et al., 2020). In addition, a different study has also suggested that DELLA ubiquitination and degradation can be induced by the E3 ubiquitin ligase FLAVIN‐BINDING KELCH REPEAT F‐BOX1 (*At*FKF1) to promote flowering under long‐day conditions (Yan et al., 2020). However, the involvement of *At*FKF1 in direct polyubiquitination of DELLA proteins has not been confirmed by in vitro assays as in Blanco‐Touriñán, Legris, et al. (2020), neither has it been demonstrated that the mechanism acts in a GA/GID1‐independent manner, for example, by showing whether *At*FKF1 affects the stability of *At*gai‐1 or *At*rgaΔ17.

While all DELLA PTMs have been extensively characterized in *Arabidopsis* and few other flowering plants, their relevance within nonflowering plant DELLAs is currently unknown. In the case of SCF^SLY1/GID2^‐induced polyubiquitination, although lycophyte and fern DELLAs can be degraded in a GA‐dependent manner (Tanaka et al., 2014; Yasumura et al., 2007) and species belonging to these plant lineages possess *At*SLY1 homologs (Hernández‐García et al., 2019), fern or lycophyte DELLA polyubiquitination has not yet been experimentally confirmed.

In bryophytes, it appears that only liverworts have *At*SLY1 homologs (Hernández‐García et al., 2019), but it is currently unknown if these homologs can induce DELLA polyubiquitination and degradation. Even more intriguing is the case of mosses and hornworts, which appear to lack *At*SLY1 homologs (Hernández‐García et al., 2019), and therefore, if DELLA polyubiquitination is present, it is mediated by other proteins. As bryophytes do not synthesize GAs (Hernández‐García et al., 2020) and *At*COP1 can induce DELLA polyubiquitination in a GA‐independent manner in *Arabidopsis* (Blanco‐Touriñán, Legris, et al., 2020), *At*COP1 orthologs might be good candidates for bryophyte DELLA polyubiquitination. Bryophytes, such as *Physcomitrella patens* (now *Physcomitrium patens*; Rensing et al., 2020), have orthologs of *At*COP1 (Ranjan et al., 2014). It would be interesting to test whether *Pp*COP1 proteins can interact with *Pp*DELLA and induce polyubiquitination, to investigate whether this important PTM is conserved in bryophytes and infer whether it was a property of the ancestral DELLA protein that was maintained during evolution.

### 5.3 DELLA downstream transcriptional targets

Flowering plant DELLA proteins have numerous and diverse transcriptional targets. Transcriptomic analyses using overexpression of DELLA proteins or mutants in GA biosynthesis or signaling (reviewed in Locascio et al., 2013b) have been carried out to elucidate the mechanisms by which DELLAs repress GA responses. Cao et al. (2006) used microarray hybridization in a quadruple *della* mutant line in an *Atga1‐3* (GA biosynthesis) mutant background (*Atgai‐t6 Atrga‐t2 Atrgl1‐1 Atrgl2‐1 Atga1‐3*) and compared gene expression with an *Atga1‐3* mutant line, to identify DELLA‐induced transcriptional changes occurring in imbibed seeds and unopened flower buds. As germination in *Arabidopsis* is regulated by *At*RGL2 primarily, as well as *At*GAI and *At*RGA1, and flowering by *At*RGL1, *At*RGL2, and *At*RGA1, the choice of the quadruple knockout should have been sufficient to enable identification of gene targets regulated by DELLAs during these developmental stages (Cao et al., 2006).

Collectively, transcriptional changes were observed in the expression of genes involved in cell growth and cell wall loosening, such as pectinesterases and expansins (most of which were repressed by DELLA), genes involved in protein phosphorylation, genes encoding transcription factors belonging to the MYB, bHLH, WRKY, and MADS‐box families, and genes regulating responses to disease, stress, and hormones (Cao et al., 2006). Some overlap was observed between DELLA‐induced gene expression in imbibed seeds and unopened flower buds, but a significant amount of transcriptional changes were tissue‐specific, suggesting that DELLA function is tightly linked to its expression patterns in the different tissues (Cao et al., 2006; Gallego‐Bartolomé et al., 2010).

Zentella et al. (2007) attempted to identify direct DELLA targets using microarray analysis after treating *Atga1‐3* seedlings with GA and thus inducing rapid degradation of all DELLAs. In addition, in the same genetic background, they overexpressed *At*rgaΔ17, which lacks the DELLA domain and is resistant to GA‐induced degradation, using a dexamethasone (DEX)‐inducible system. This experiment stimulated rapid and high induction of stable *At*RGA1, aiming to identify early transcriptional changes that are more likely to be directly induced by *At*RGA1 (Zentella et al., 2007). Among the genes that were differentially expressed in both data sets were GA biosynthesis and perception genes, such as *AtGID1* and GA‐oxidase‐encoding genes, nuclear transcription factors or regulators, such as *bHLH*s, *MYB*s, *WRKY27*, and *SCR‐LIKE3* (*AtSCL3*), as well as genes encoding E2 conjugating enzymes and E3 ligases, such as *AtXERICO*, which is activated by DELLA to induce abscisic acid (ABA) biosynthesis (Zentella et al., 2007). All these genes were induced by *At*rgaΔ17 and repressed by GA.

Early transcriptional responses to DELLA induction were also identified by Gallego‐Bartolomé et al. (2011) using etiolated seedlings overexpressing either *At*gai‐1 under the control of a heat‐shock promoter or a translational fusion between *At*gai‐1 and the glucocorticoid receptor (GR) domain under the control of the *AtGAI1* promoter. Activation of expression by either heat‐shock induction or treatment with DEX and cycloheximide (CHX), led to the identification of early transcriptional targets involved in processes such as GA homeostasis, stress responses, and hormone signaling and biosynthesis (Gallego‐Bartolomé et al., 2011). Most notably, the promoters of DELLA downstream target genes were statistically enriched in the cis elements recognized by transcription factors that were later shown to interact with DELLA proteins, such as DNA BINDING1 ZINC FINGER6 (*At*DOF6), regulating seed dormancy (Ravindran et al., 2017) and ARABIDOPSIS RESPONSE REGULATOR1 (*At*ARR1) regulating root meristem identity (Marín‐de la Rosa et al., 2015; see Section 5.4).

A subsequent meta‐analysis of transcriptomic data sets by Locascio et al. (2013b) identified genes involved in GA metabolism to be regulated by DELLAs under most physiological contexts in most tissue types. This confirms the previously described role of DELLAs in regulating the feedback response to maintain GA homeostasis (Dill & Sun, 2001; Itoh et al., 2002; Wen & Chang, 2002). Furthermore, transcriptomic analyses have demonstrated that DELLA proteins exert their repression on plant size by interfering with two main cellular processes: cell expansion, by regulating cell wall biogenesis and modification, and cell division, by regulating cell‐cycle genes (Locascio et al., 2013b).

Many of the flowering plant DELLA target genes regulate processes conserved in nonflowering plants, such as cell wall biogenesis (e.g., Shibaya & Sugawara, 2007) and cell cycle regulation (e.g., Nishihama et al., 2015). Therefore, future studies should investigate the putative role of DELLA proteins in these processes using nonflowering plant model species. In addition, comparative transcriptomic analyses in land plants from different lineages, in response to induction or impairment of DELLA signaling, will provide more insights into whether DELLA transcriptional targets are conserved in land plants.

### 5.4 DELLA protein–protein interactions

Several studies have been carried out to understand the mechanisms by which the observed transcriptional changes are brought about by DELLAs in flowering plants. Attempts have been made to identify direct DELLA binding to gene promoters via chromatin immunoprecipitation (ChIP); however, DELLAs have not shown any direct DNA binding (Feng et al., 2008). Instead, it was demonstrated that DELLA protein function relies on protein–protein interactions having direct or indirect effects on transcription (e.g., de Lucas et al., 2008; Feng et al., 2008; Hou et al., 2010). Marín‐de la Rosa et al. (2014) carried out a large yeast‐two hybrid screen using the GRAS domain of *At*GAI and identified 57 unique transcription factors as DELLA interactors. These belonged to 15 different transcription factor families regulating a big range of plant growth responses, including germination, vegetative growth, reproductive development, light signaling, stress responses, and hormone signaling. However, this screen did not identify all the putative DELLA protein interactors, as the *Arabidopsis* transcription factor library used was only ~75% complete and a truncated version of DELLA, containing only the GRAS domain, was used as bait (Marín‐de la Rosa et al., 2014).

A more recent study used truncated versions of *At*RGA1 and *At*GAI1 (containing the GRAS domain) as bait to screen a library of 1956 *Arabidopsis* transcriptional regulators for DELLA interaction using yeast‐two hybrid (Lantzouni et al., 2020). *At*RGA1 and *At*GAI1 interactor sets showed ~87% overlap, supporting the hypothesis that DELLA function is tightly linked to its expression patterns in the different tissues rather than its ability to interact with different transcription factors (Gallego‐Bartolomé et al., 2010; Lantzouni et al., 2020). Furthermore, the screen identified more than 250 DELLA interaction partners, raising the total number of putative DELLA interactors to more than 350 (Lantzouni et al., 2020).

The vast majority of DELLA interactions regulate transcription; however, there are few cases where they also regulate other processes, for example, microtubule organization (Locascio et al., 2013a). The four main mechanisms by which DELLA interactions regulate transcription in flowering plants are outlined in the sections below.

#### 5.4.1 Sequestration of transcription factors and chromatin remodeling factors

**Figure 4:**
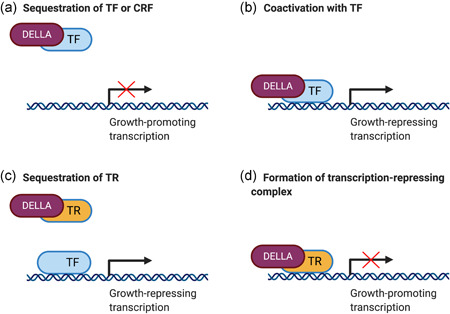
The four main mechanisms by which DELLA protein–protein interactions regulate transcription. (a) DELLA proteins sequester transcription factors (TFs) or chromatin remodeling factors (CRFs) to prevent activation of growth‐promoting transcription. (b) DELLA proteins bind to TFs and coactivate growth repressing transcription. (c) DELLA proteins sequester transcriptional regulators (TRs) that repress TFs, thus promoting growth‐repressing transcription. (d) DELLA proteins can form complexes with TRs which inhibit growth‐promoting transcription (figure created with BioRender.com) [Color figure can be viewed at wileyonlinelibrary.com]

The majority of DELLA protein–protein interactions characterized to date in flowering plants involve the sequestration of transcription factors, often those that promote growth, thus preventing activation of their downstream target genes (Figure 4). The PHYTOCHROME‐INTERACTING FACTORS (*At*PIFs) were the first transcription factors identified to be repressed via interaction with DELLA proteins, establishing a mechanism by which flowering plants are able to integrate light and GA signaling to regulate hypocotyl elongation (de Lucas et al., 2008; Feng et al., 2008). According to the characterized mechanism, light activates phytochrome photoreceptors, which induce *At*PIF phosphorylation and subsequent degradation via the 26S proteasome, preventing *At*PIF‐activated hypocotyl elongation (Al‐Sady et al., 2006; E. Park et al., 2004). At the same time, light induces a reduction in GA levels which stabilizes DELLA proteins (Achard et al., 2007) and allows them to interact with the DNA‐binding bHLH domain of *At*PIFs (an interaction that requires the LHR1 domain of DELLA; Figure 3), forming an inactive complex (de Lucas et al., 2008; Feng et al., 2008). Interaction with DELLA proteins prevents *At*PIFs from binding to G‐box elements on promoters of target genes such as *β‐EXPANSIN* and *LIPID TRANSFER PROTEIN3* (*AtLTP3*), which promote GA‐induced etiolation in darkness (skotomorphogenesis; de Lucas et al., 2008).

In addition to transcription factors, DELLA proteins can also sequester chromatin remodeling factors to inhibit GA responses. Zhang et al. (2014) demonstrated that DELLAs interact with the chromatin remodeling factor PICKLE (*At*PKL) to prevent the latter from binding to transcription factors, such as *At*PIF3 and BRASSINAZOLE‐RESISTANT 1 (*At*BZR1), and promoting histone H3 lysine‐27 trimethylation (H3K27me3) on promoters of target genes regulating GA‐ and brassinosteroid‐induced hypocotyl elongation.

The transcription factor sequestration mechanism has also been observed in rice, another flowering plant, where the DELLA protein *Os*SLR1 interacts with NO APICAL MERISTEM 29 (*Os*NAC29) and *Os*NAC31 to prevent binding to *Os*NAC targets such as *OsMYB61*, which induces *CELLULOSE SYNTHASE* (*OsCESA*) genes that promote secondary wall cellulose synthesis required for internode development (Huang et al., 2015). Whether the DELLA sequestration mechanism is present outside flowering plants remains elusive. Bryophytes do have orthologs of proteins such as the *At*PIFs (Inoue et al., 2016; Possart et al., 2017); therefore, it would be interesting to test whether the DELLA–PIF interaction and the sequestration mechanism is present in bryophyte model species too, to elucidate whether the mechanism is universal in the land plant phylogeny.

#### 5.4.2 Coactivation with transcription factors or regulators

A different mechanism by which DELLAs interact with other proteins is by acting as transcriptional coactivators, activating growth repressing transcription (Figure 4). For example, DELLAs bind ABSCISIC ACID INSENSITIVE 3 (*At*ABI3) and *At*ABI5 and coactivate transcription of *SOMNUS* (*AtSOM*), which induces ABA biosynthesis and represses GA biosynthesis, forming a positive feedback loop to inhibit seed germination at high temperatures (Lim et al., 2013). DELLAs can also interact with the transactivation domain of *At*ARR1, enhancing the transactivation of cytokinin‐regulated *At*ARR1 targets, reducing the rate of cell division in the root meristem to maintain meristem identity (Marín‐de la Rosa et al., 2015).

DELLA coactivation of gene expression has also been reported in *Medicago truncatula*, a eudicot flowering plant model species, which forms symbiotic relationships with the nitrogen‐fixing bacteria of the genus *Rhizobium. Mt*DELLA proteins can interact with the transcription factors NODULATION SIGNALING PATHWAY2 (*Mt*NSP2) and *Mt*NF‐YA1 to coactivate the expression of *ETHYLENE RESPONSIVE FACTOR REQUIRED FOR NODULATION1* (*MtERN1*), which induces downstream gene expression required for the progression of rhizobial infections (Fonouni‐Farde et al., 2016).

As the DELLA transactivation ability is conserved at least in members of lycophytes and all three bryophyte groups (Hernández‐García et al., 2019), this mechanism of interaction could have been a property of the ancestral DELLA protein. This should be further examined in the future, for example, by identifying DELLA downstream targets that are downregulated in the transcriptome of bryophyte *della* mutants, as well as by examining the interactome of those DELLA proteins, to identify and test potential interactions with transcription factors that may involve DELLA transactivation.

#### 5.4.3 Sequestration of transcriptional regulators

A third mechanism by which DELLAs interact with other proteins is by sequestering transcriptional regulators to inhibit their repressive function on growth‐repressing transcription (Figure 4). This was first demonstrated with the interaction between DELLA and JASMONATE‐ZIM‐DOMAIN PROTEIN 1 (*At*JAZ1), a negative regulator of jasmonate (JA) responses (Hou et al., 2010). Binding of DELLA to *At*JAZ1 prevents the latter from interacting with *At*MYC2 and repressing *At*MYC2‐mediated JA‐responsive gene expression regulating root development (Hou et al., 2010).

The DELLA–JAZ interaction has also been described in rice (Um et al., 2018), but it is unknown if it is conserved outside flowering plants. This can be addressed by characterizing interactions of nonflowering plant DELLA proteins. Given that bryophyte genomes, such as that of *Marchantia polymorpha*, encode orthologs of *At*JAZ1 and *At*MYC2 (Bowmanan et al., 2017), investigating the interaction of *Mp*DELLA with *Mp*JAZ and whether it regulates *MpMYC* transcription, would be a good starting point.

#### 5.4.4 Repression on transcription by complex formation

DELLA proteins are also able to repress transcriptional activation by acting as parts of transcriptional complexes and repressing growth‐promoting transcription (Figure 4). For example, DELLA requires the interaction and formation of a complex with the transcriptional regulator BOTRYTIS SUSCEPTIBLE1 INTERACTOR (*At*BOI), to inhibit GA responses such as germination, flowering, and juvenile‐to‐adulthood phase transition via binding to promoters of GA‐inducible genes such as *EXPANSIN 8* (*AtEXPA8*), *PACLOBUTRAZOL RESISTANCE 1* (*AtPRE1*), and *AtPRE5* (J. Park et al., 2013). Regarding the regulation of flowering in particular, studies have shown that the *At*BOI–DELLA complex can delay flowering by targeting the *FLOWERING LOCUS T* (*AtFT*) promoter (Nguyen et al., 2015). As *AtBOI* expression can be induced by *Botrytis cinerea* and *Pseudomonas syringae* pv. tomato DC3000 (Luo et al., 2010), it is also likely that interaction with DELLA enables the regulation of various plant phase transitions during pathogen attack.

As bryophytes, such as *P. patens*, can be infected by *B. cinerea* (Ponce de León et al., 2007) and the *P. patens* genome encodes *At*BOI homologs (Rensing et al., 2008), it would be interesting to test whether this mechanism and its relevance to pathogen attack is conserved in *P. patens* and other bryophytes.

## 6 THE EVOLUTION OF DELLA SIGNALING: A CASE OF MOLECULAR EXPLOITATION

Over the past two decades, several studies have been conducted to identify how DELLA signaling evolved. The current dogma suggests that only vascular plants possess the characterized DELLA signaling pathway regulating GA responses, and that the functionality of the mechanism components was acquired gradually during the course of land plant evolution via molecular exploitation (Hernández‐García et al., 2019; Yasumura et al., 2007). This hypothesis is supported by biochemical studies demonstrating that the only land plant groups that possess bioactive GAs are flowering plants, gymnosperms and some ferns and lycophytes (Figure 1; Aya et al., 2011; MacMillan, 2001; Tanaka et al., 2014). Consequently, this raises the question of how DELLA proteins are regulated in earlier‐diverging land plants where bioactive GAs are not present, and whether they are able to repress growth responses in a similar manner.

### 6.1 DELLA orthologs are present throughout the land plant lineage

Bioinformatic analyses using species from all three bryophyte groups, including mosses such as *P. patens* and *Sphagnum fallax*, liverworts such as *M. polymorpha*, and hornworts such as *Nothoceros vincentianus* and the recently sequenced *Anthoceros agrestis* and *Anthoceros punctatus*, have suggested that DELLA orthologs are present in all three bryophyte groups, while *At*SLY1 orthologs are only found in liverworts (Hernández‐García et al., 2019; Li et al., 2020). Despite the confirmed presence of GRAS family proteins in two charophyte families, Zygnematales and Coleochaetales, DELLA proteins appear to be absent from algae, suggesting that they evolved in the common ancestor of land plants (Figure 1; Hernández‐García et al., 2019).

GID1 homologs have been identified in bryophytes, such as GID1‐like (*Pp*GLPs) in *P. patens*; however, these proteins lack the defining features of true flowering plant GID1s, such as the catalytic triad forming the GA pocket or the N‐terminal lid required for interaction with DELLA (Figure 2; Hirano et al., 2007). Similarly, these features are absent in *Mp*GLPs, suggesting that *M. polymorpha* does not possess canonical GA signaling, despite the presence of an *AtSLY* ortholog (Hernández‐García et al., 2019). It appears that *At*GID1 orthologs are exclusively found in vascular plants, including ferns and lycophytes such as *Selaginella moellendorffii*, although partial sequences from bryophytes such as *Phaeoceros carolinianus* and *Paraphymatoceros halli* suggest that GID1 orthologs may be present in some hornworts (Hernández‐García et al., 2019), the group that diverged earliest in the bryophyte lineages (Li et al., 2020). It would be interesting to examine whether these putative hornwort GID1 orthologs possess the biochemical properties of true GID1s, to provide more evidence for the presence or absence of GID1 orthologs from bryophytes.

Despite the fact that bryophyte DELLAs have a highly conserved GRAS domain, the N‐terminal DELLA domain, which is necessary for interaction with GID1, is more divergent in mosses such as *P. patens* (Hernández‐García et al., 2019; Hirano et al., 2007). Interestingly, this is not the case for a number of hornworts, including *N. vincentianus* and *Anthoceros* species, which have DELLAs with highly conserved N‐terminal domains, a number of liverworts, including *M. polymorpha*, as well as other mosses, such as *Takakia lepidozioides*, whose DELLA protein has a highly conserved DELLA and VHYNP motif, but lacks the LEQLE motif within the wider DELLA domain (Hernández‐García et al., 2019; Li et al., 2020). Ancestral protein reconstruction has also suggested that the predicted ancestral DELLA peptide sequence displays a highly conserved N‐terminal domain (Hernández‐García et al., 2019). These observations imply that the ancestral DELLA, as well as a number of bryophyte DELLAs, were probably already equipped for interaction with GID1 homologs (Hernández‐García et al., 2019).

Following from the above observations, it is unclear why the majority of mosses studied so far display a more divergent N‐terminal domain and what selective forces might have brought about those amino acid changes. Interestingly, a similar pattern of peptide sequence conservation has been observed in DELLA3 clade proteins in rice, such as *Os*SLR1‐like (*Os*SLRL1), which has lost the DELLA domain, but is still able to induce dwarfism and remain stable in the presence of GA (Itoh, Shimada, et al., 2005). This observation implied that moss DELLAs with a divergent N‐terminal domain such as *Pp*DELLAs might still be able to repress growth in a GA‐independent manner (see Section 6.3). In silico comparative gene co‐expression network analysis using putative orthologs of DELLA‐interacting transcription factors in *Arabidopsis*, tomato (two flowering plants), *P. patens* (bryophyte), and *Chlamydomonas reinhardtii* (green alga), suggested that the function of *Pp*DELLAs was most likely to regulate stress responses, and that coordination between the functions regulated by DELLAs increased during the course of evolution (Briones‐Moreno et al., 2017). This suggests that bryophyte DELLAs may have been able to repress growth in response to stress in a GA‐independent manner, although this hypothesis awaits experimental confirmation.

It is also interesting to note that bryophyte genomes encode the enzymes catalyzing the first committed biochemical reactions involved in GA biosynthesis, such as *ent*‐copalyl diphosphate synthase (CPS) and *ent*‐kaurene synthase (KS)—although bryophytes possess bifunctional enzymes (CPS/KS)—that catalyze the conversion of *trans*‐geranylgeranyl diphosphate (GGPP) into *ent*‐kaurene, as well as *ent*‐kaurene oxidase (KO), which oxidizes *ent*‐kaurene into *ent*‐kaurenoic acid (Bowmanan et al., 2017; Hayashi et al., 2010; Li et al., 2020). In addition, hornworts and liverworts have one more enzyme required for GA biosynthesis, *ent*‐kaurenoic acid oxidase (KAO), which is not encoded by the *P. patens* or *S. fallax* genomes (Li et al., 2020). Nevertheless, it appears that bryophytes lack orthologs of GA20ox and GA3ox, required for the biosynthesis of bioactive GAs, or GA2ox, required for GA catabolism, in contrast to vascular plants, including lycophytes and ferns, where the complete biosynthesis pathway can be found (Hernández‐García et al., 2020; Li et al., 2020; Tanaka et al., 2014). Whether the endogenous diterpenes found in bryophytes have a role in DELLA signaling remains elusive (see Section 6.4).

### 6.2 The GID1‐binding ability of DELLAs was most likely present in bryophytes

Several attempts have been made to test whether bryophyte or lycophyte DELLA and GID1 homologs are able to interact in a GA‐dependent manner. Yeast two‐hybrid assays demonstrated that *Sm*DELLAs could interact with *Sm*GID1s in a GA‐dependent manner and this was further supported by in vitro binding assays showing that *Sm*GID1 proteins could bind GA_4_ in the presence of *Sm*DELLA1 (Hirano et al., 2007). Similarly, Yasumura et al. (2007) demonstrated that proteins from a different lycophyte, *Selaginella kraussiana, Sk*GID1 and *Sk*DELLA, could interact in yeast cells in the absence of GAs, but much more strongly in the presence of GA_3_, suggesting that canonical GA signaling is present in lycophytes.

In contrast, homologous proteins in moss, *Pp*GLP1 and *Pp*DELLAs, were not able to interact in the presence or absence of GAs, and *Pp*GLP1 could not bind GA_4_ or other GAs in vitro in either the presence or absence of *Sm*DELLA1 (Hirano et al., 2007; Yasumura et al., 2007). Interestingly, *Pp*GLP1 was able to interact with *Sk*DELLA in the presence of GA_3_, however, the interaction of similar magnitude was also observed in the absence of GA_3_, indicating that the interaction was GA‐independent (Yasumura et al., 2007). This finding was not supported by Hirano et al. (2007) who observed that *Pp*GLP1 could not interact with DELLAs from a different *Selaginella* species, *S. moellendorffii*. Furthermore, *Pp*DELLAs were not able to interact with any GID1 homolog (Hirano et al., 2007). These observations suggested that bryophyte GLPs probably possessed an affinity for DELLAs that was maintained during GID1 evolution—although this was only supported by the observation that *Pp*GLP1 could interact with *Sk*DELLA—whereas DELLA affinity for GID1 most likely arose after the bryophyte divergence (Yasumura et al., 2007).

This hypothesis was later challenged by Hernández‐García et al. (2019) who demonstrated that, while *MpDELLA, PpDELLAa*, and the DELLA from the moss *T. lepidozioides* (*Tl*DELLA) could not interact with *At*GID1s in yeast cells in a GA‐dependent manner, DELLA from the hornwort *N. vincentianus* was able to interact with *At*GID1s in a GA‐dependent manner, suggesting that DELLA affinity for GID1 homologs may have evolved as early as the hornwort divergence. As hornworts appear to have diverged earliest in the bryophyte lineages (Li et al., 2020), it is possible that the ancestral land plant DELLA probably possessed GID1 affinity and it was later lost in mosses and liverworts. Furthermore, the fact that *Mp*DELLA or *Tl*DELLA have fairly conserved N‐terminal domains but are still unable to interact with *At*GID1s, suggests that conservation of the DELLA N‐terminal domains is not sufficient for interaction with GID1 homologs and that conservation of other regions might be necessary to enable this interaction (Hernández‐García et al., 2019). It is also important to note that the *Nv*DELLA‐*At*GID1 interaction as well as all other interactions described in this section have only been tested in the yeast two‐hybrid system, and therefore, further in vivo interaction assays will need to be carried out to confirm these findings, before drawing any major conclusions.

In addition, yeast two‐hybrid assays by Yasumura et al. (2007) showed that *Sk*DELLA was also able to interact with *At*GID1c in a GA‐dependent manner, whereas *At*RGA1 was not able to interact with *Sk*GID1 at all. This led to the conclusion that DELLA specificity for GID1 became tighter during the course of evolution (Yasumura et al., 2007). Measurements of β‐galactosidase activity have also indicated that the DELLA–GID1 interaction in *Arabidopsis* is much more GA‐dependent than in *S. kraussiana*, suggesting that GA potentiation increased with land plant evolution (Yasumura et al., 2007). This hypothesis was further supported by biochemical studies showing that GID1 affinity for bioactive GAs increased with land plant evolution (Hirano et al., 2007; Yoshida et al., 2018). In addition, studies using the fern *Lygodium japonicum* have shown that minute concentrations of GA_4_ enable *Lj*GID1 and *Lj*DELLA proteins to interact in yeast cells and that GID1 affinity for GA_4_ is much greater than that of seed plant GID1s, suggesting an increase in GA potentiation in the ancestor of ferns (Tanaka et al., 2014). The exceptional affinity of *Lj*GID1 for GA_4_, in this case, is probably a consequence of the very specific function of GA_4_ (sex determination) in a very specific tissue type (young prothalli), where selection would favor tight specificity of *Lj*GID1 for GA_4_ to ensure proper sex organ development.

### 6.3 DELLAs were co‐opted to regulate growth repression in flowering plants

Complementation assays have shown that *Sm*GID1s were able to complement the function of *Os*GID1 in the *Osgid1‐3* mutant and *Sm*DELLAs were able to repress growth in wild‐type rice, whereas *P. patens* homologs could not (Hirano et al., 2007). In contrast, overexpression of *Pp*DELLAa–GFP in the *Arabidopsis* slender *Atgai‐t6 Atrga‐24 Atga1‐3* mutant induced dwarfism (Yasumura et al., 2007). The discrepancy between the two observations on the effect of *Pp*DELLA overexpression on growth in rice and *Arabidopsis* has been attributed to the fact that wild‐type rice was used in one study, where *Os*SLR1 was still actively suppressing growth responses, whereas the *Arabidopsis* line in the other study was a double *della* mutant in a GA‐deficient background, and thus DELLA‐induced vegetative growth suppression had already been eliminated (Hirano et al., 2007).

Application of GA_3_ to *Arabidopsis* plants overexpressing *pRGA::GFP‐SkDELLA* resulted in the loss of the fluorescence signal, presumably due to GA_3_‐induced degradation of *Sk*DELLA, whereas loss of fluorescence was not observed when plants overexpressing *pRGA::GFP‐Pp*DELLAa were treated with GA_3_ (Yasumura et al., 2007). These observations support the hypothesis that bryophyte DELLAs have the capacity to induce growth repression in a GA‐independent manner. Furthermore, as has been pointed out by Yasumura et al. (2007), it is highly likely that *Pp*DELLA was able to induce vegetative growth repression in *Arabidopsis* and not in *P. patens*, because downstream gene expression regulating growth has evolved the ability to respond to DELLA proteins in flowering plants, but not in mosses. This supports a model of evolution where, after the emergence of GAs in vascular plants, DELLA proteins, which already existed as transactivation‐inducing proteins, were co‐opted to regulate growth repression in a GA‐dependent manner.

### 6.4 *P. patens* possesses a diterpene signaling mechanism which might be uncoupled from DELLA signaling

Experiments have provided evidence that a putative GA‐like/diterpene signaling pathway is present in mosses. As pointed out earlier, *P. patens* possesses GA signaling and biosynthesis orthologs such as *Pp*DELLAs, *Pp*CPS/KS, and *Pp*KO, and produces the diterpenes *ent*‐kaurene, *ent*‐kaurenoic acid, and the recently discovered *ent*‐3β‐hydroxy‐kaurenoic acid (3OH‐KA; Hayashi et al., 2006, 2010; Miyazaki et al., 2018). *Ppdella* mutants do not display any obvious defects in vegetative growth (Yasumura et al., 2007); however, further analysis is necessary to establish if they produce phenotypes at different developmental points that have been overlooked.

Disruption of *PpCPS*/*KS* results in the suppression of chloronema to caulonema differentiation, required for normal vegetative growth in *P. patens*, and the phenotype can be rescued upon exogenous application of *ent*‐kaurene or *ent*‐kaurenoic acid, which are naturally synthesized by *P. patens*, as well as by application of the fern antheridiogen GA_9_ methyl‐ester (GA_9_‐Me; Hayashi et al., 2010; Tanaka et al., 2014). In addition, loss of *PpCPS*/*KS* in *P. patens* results in a decrease in the rate of spore germination, a phenotype that can be partially rescued by application of exogenous *ent*‐kaurene or GA_9_‐Me, as well as a decrease in total dry weight when grown in liquid cultures (Pan et al., 2015; Vesty et al., 2016).

Wild‐type *P. patens* is also responsive to the exogenous application of diterpenes. Application of *ent*‐kaurene to moss protonemata results in increased production of caulonemata as well as a faster spore germination rate (Hayashi et al., 2010; Vesty et al., 2016). A similarly faster germination rate is also induced upon application of exogenous GA_9_‐Me on wild‐type moss spores (Vesty et al., 2016). In the case of *S. moellendorffii*, exogenous application of GA_4_ induces an increase in outer exospore projection heights in microspores, demonstrating GA bioactivity (Aya et al., 2011). Uniconazole, which inhibits the conversion of *ent*‐kaurene into *ent*‐kaurenoic acid, induces growth repression in *S. moellendorffii* and produces defects in microspore outer exospore walls; however, only the latter can be rescued by exogenous application of GA_4_ (Aya et al., 2011; Hirano et al., 2007). Similarly, in *P. patens*, paclobutrazol (PAC), which also inhibits the biosynthesis of *ent*‐kaurenoic acid, induces a growth phenotype that cannot be rescued by exogenous application of GA_3_, suggesting that a diterpene signaling pathway regulating growth exists in *P. patens* (Yasumura et al., 2007). This pathway is probably uncoupled from DELLA signaling, as the *PpdellaAB* mutant does not display faster vegetative growth and is sensitive to exogenous application of PAC at the vegetative stage (Yasumura et al., 2007).

As mentioned earlier, overexpression of *PpDELLAa* driven by the *AtRGA1* promoter in the *Arabidopsis* slender *Atgai‐t6 Atrga‐24 Atga1‐3* mutant induces dwarfism, demonstrating that *Pp*DELLAs possess the ability to inhibit growth in *Arabidopsis* (Yasumura et al., 2007). It has also demonstrated both in yeast and *Nicotiana benthamiana* that the N‐terminal domain of *PpDELLAa*, as well as other bryophyte DELLAs, possesses the ability to induce transactivation, as mentioned earlier, despite being more divergent compared with other bryophyte DELLAs, suggesting that *Pp*DELLAs may share functional homology with vascular plant DELLAs (Hernández‐García et al., 2019).

Collectively, these observations suggest that a diterpene signaling mechanism involving a molecule similar to GA_9_‐Me is present in *P. patens* regulating germination and morphogenesis. The recently identified 3OH‐KA is suggested to be the end‐product of the moss diterpene biosynthesis pathway and its exogenous application can rescue the defects in caulonemal differentiation observed in the moss *Ppcps*/*ks* mutant (Miyazaki et al., 2018). It would, therefore, be interesting to test if 3OH‐KA can potentiate the interaction between *Pp*GLPs and *Pp*DELLAs or induce *Pp*DELLA degradation. The absence of a GID1 ortholog in *P. patens* makes it unlikely that moss diterpene and *Pp*DELLA signaling are linked; however, it cannot be ruled out that *P. patens* has a completely novel receptor for perceiving the bioactive diterpene. More detailed characterization of *Ppdella* mutants, as well as more in vivo interaction assays in P. patens and other bryophytes, will be necessary to shed more light on the evolution of DELLA signaling in *P. patens* and other bryophytes.

### 6.5 Concluding remarks

DELLA proteins originated in land plants (Hernández‐García et al., 2019). It is not clear whether they could induce growth repression within their species, but they could induce transactivation via their N‐terminal domain, suggesting that they might have already been functioning as transcriptional “hubs” (Hernández‐García et al., 2019; Yasumura et al., 2007). Although some bryophyte DELLAs, such as the hornwort *Nv*DELLA, can bind *At*GID1s in a GA‐dependent manner, this property is absent from the majority of bryophyte DELLAs examined so far (Hernández‐García et al., 2019). Whether GA‐dependent *At*GID1 interaction is universal among hornwort DELLA orthologs remains elusive.

Intriguingly, *P. patens* possesses a diterpene signaling pathway, but it is unclear whether this pathway is linked in any way with the *Pp*DELLA signaling pathway (Hayashi et al., 2010; Yasumura et al., 2007). It would be useful to investigate whether any other bryophytes have similar diterpene signaling pathways regulating growth responses and whether DELLA proteins are involved in those pathways. In addition, *M. polymorpha* and other liverworts appear to be the only bryophytes that have an *At*SLY1 ortholog (Hernández‐García et al., 2019). Thus, it would be interesting to examine whether *Mp*DELLA is linked in any way with *Mp*SLY1, for example, by investigating the stability of *Mp*DELLA in an *Mpsly1* mutant.

Collectively, the evidence so far suggests that canonical GA signaling involving DELLA, GID1, and SLY1/GID2 proteins appeared with the evolution of vascular plants, where bioactive GAs first appeared, exploiting the transactivation domain of DELLA proteins to enable DELLA interaction with the GA–GID1 complex (Figure 1; Hernández‐García et al., 2019). As DELLA signaling predates GA signaling, it is likely that GAs exploited the already established DELLA signaling mechanisms to control growth‐regulating transcription (Hernández‐García et al., 2019), and DELLA functions were refined in different species according to their expression patterns (Gallego‐Bartolomé et al., 2010) and perhaps by PTMs too.

Molecular exploitation appears to be a common mechanism driving the evolution of hormone signaling across kingdoms. A very well‐known example of this phenomenon is the evolution of steroid hormone receptors in vertebrates (Eick & Thornton, 2011). The biosynthetic pathway of estrogens (female sex steroid hormones) involves the production of testosterone and progesterone as precursors (Hanukoglu, 1992). Interestingly, nuclear receptors specific for estrogens evolved first, and receptors with affinities for testosterone and progesterone diverged later, exploiting steroid precursors that were already present (Thornton, 2001).

In addition, it has been shown that the mineralocorticoid receptor (MR) of the steroid hormone aldosterone, regulating electrolyte homeostasis, evolved an affinity for the hormone before aldosterone had actually emerged (Bridgham et al., 2006). In fact, the ancestral receptor had an affinity for structurally similar steroids that appeared early in vertebrate evolution, and this affinity was later exploited by aldosterone, which emerged more recently in the ancestor of tetrapods, establishing a tetrapod‐specific MR–aldosterone partnership with a novel function (Bridgham et al., 2006). Similarly, in vascular plants, the DELLA N‐terminal domain regulating transactivation was exploited by the GA–GID1 complex for interaction, recruiting DELLA signaling into GA signaling (Hernández‐García et al., 2019). These examples demonstrate that across kingdoms, novel interactions can evolve when newly emerged small molecules or proteins are co‐opted to interact with pre‐existing modules. This enables the development of novel functions and adds to the complexity of signaling pathways (Bridgham et al., 2006). In the case of DELLA proteins, it would be useful to test whether bryophyte DELLA proteins are able to interact with homologs of the numerous flowering plant DELLA interacting partners to confirm that DELLAs possessed this ability before the evolution of vascular plants.

The question of how DELLA proteins arose in the very first land plants, and what the selection pressures were that retained them during early land plant evolution, still remains unanswered. In silico comparative gene co‐expression network analysis has suggested the hypothesis that the function of *Pp*DELLAs, and by extension early land plant DELLAs, was most likely to regulate stress responses (Briones‐Moreno et al., 2017). To validate this hypothesis, it is necessary to test how the *PpdellaAB* mutant performs under various forms of stress. Experiments by Yasumura et al. (2007) have shown that *PpdellaAB* is sensitive to salt stress. Whether this is the case for other bryophytes or other forms of stresses is unknown. Future studies should concentrate on using bryophyte species with sequenced genomes as well as other emerging model species, to carry out more in vivo genetic and biochemical studies to shed more light on the evolution of DELLA signaling in land plants. Analysis of additional bryophyte and charophyte genomes or transcriptomes may pinpoint the emergence of DELLAs more accurately.

Understanding how DELLA signaling mechanisms have evolved and how DELLAs respond to stress and other environmental signals could enable us to engineer better crops, to contribute to mitigating the effects of global warming, and achieving global food security. As DELLA proteins function via protein–protein interactions, targeting their interaction capacity by either identifying novel *DELLA* alleles or by manipulating some of the DELLA interaction partners could be a potential avenue for enabling production or breeding of a new generation of resilient land plants.

## CONFLICT OF INTERESTS

The authors declare that there are no conflict of interests.
